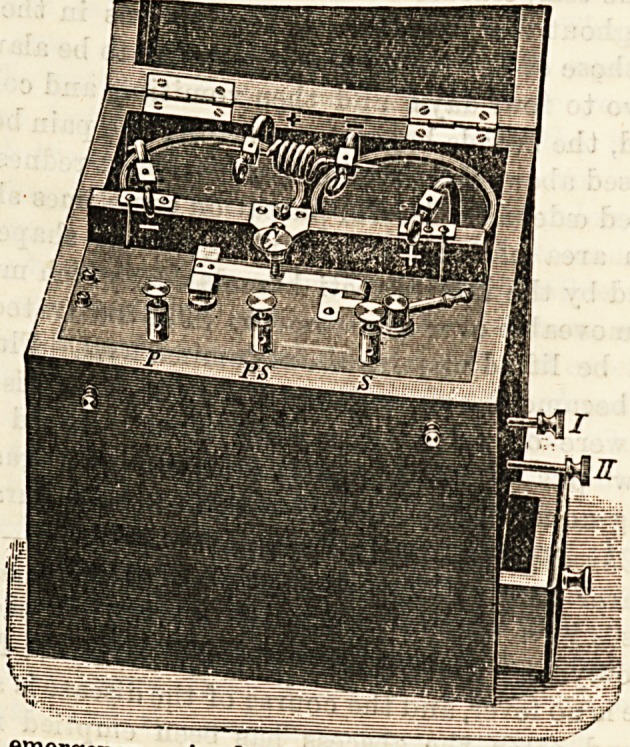# New Appliances and Things Medical

**Published:** 1894-10-13

**Authors:** 


### NEW APPLIANCES AND THINGS MEDICAL.

### THE ACME PORTABLE DRY CELL BATTERY.

(Hockin, Wilson, and Co., 13 to 16, New Inn Yard, Tottenham Court Road, W.)

The above firm have lately forwarded us a specimen of their new dry cell. This form of battery has many obvious advantages over the usual fluid-celled variety. In the first place, it is always ready for use; and secondly, it can be safely carried about from place to place without danger of the contents escaping from the cells, and possibly damaging the metal portions of the mechanism. Although the battery is primarily intended to induce the Faradic, or interrupted current, it is of sufficiently high electro-motive power to be employed as a constant current in electro-therapeutics. And the use of the battery is so simple, and the method of changing from the constant to the interrupted form of current so easy, that even in the most inexperienced hands it is almost impossible that a mistake can take place. The battery will last, without refilling the cells, from one to two years, according to the frequency of use, and at the end of that time can be supplied with new cells at a trifling cost. The entire battery, containing two cells fitted in a strong oak case and supplied with two handles and wires, brush, flat and round electrodes, may be purchased for £2 10s., a price which is often spent in repairing a more complicated form of battery. It is a variety which we strongly recommend to the notice of hospital authorities, since in the hands of students or other inexperienced persons it is not likely to suffer damage, or be put out of order, and consequently it will be found ready for us, on an emergency. A desideratum seldom experienced in hospital administration.

## Figures and Tables

**Figure f1:**